# Appendicitis in Children.—II

**Published:** 1911-04-08

**Authors:** O. L. Addison


### Hospital Clinics.

### APPENDICITIS IN CHILDREN.—II.

By O. L. ADDISON, F.R.C.S.

(A Lecture delivered at the Hospital for Sick Children, Great Ormond Street, W.C.)

In a mild case recovery takes place in from three to four days, and the restoration to health may be apparently perfect, though it is more usual for colicky pains or a feeling of discomfort to occur at intervals until a second attack supervenes. It is most unlikely that the appendix itself is ever restored to an absolutely normal condition. Some pathological condition usually results in the form of obstruction to the lumen, with consequent later dilatation of the distal portion, adhesions binding the appendix down, or causing kinks. Any of these conditions renders the appendix very liable to another acute attack.

When an abscess forms, the prognosis is far more serious. If not operated on, a fatal result is usual from toxæmia, general peritonitis, pylephlebitis, etc., though a cure, temporary or permanent, may result from rupture of the abscess into the bowel, the common situations for this to occur being the cæcum, ascending colon, or rectum. Sometimes the abscess becomes adherent to the anterior abdominal wall, and after tracking about among the muscles finally makes its way to the surface, but this method of drainage is seldom sufficiently free to allow of the abscess drying up, and more frequently results in a permanent sinus, with pocketing and further abscesses. A small abscess of a not very virulent nature may become semi-inspissated and sterile, and remain quiescent for a time, though likely to flare up at any moment. Abscesses may spread in any direction or rupture into any viscus, *e.g.* the bladder, when an acute cystitis results, with a persisting bacillus coli bacilluria, should the child recover. Spread of the abscess into the substance of the ilio-psoas is an extremely unfavourable event, owing to the difficulty of adequate drainage. Perinephritic and subphrenic abscesses and empyema all occur. Multiple liver abscesses, the result of infection *viâ* the portal vein, are invariably fatal. In two instances I have met a single abscess in the liver, both probably direct infections from perinephritic abscesses, and in both cases a cure was effected by operation. General peritonitis resulting from the rupture of an abscess is fatal unless operated on early.

### Treatment.

The rules of treatment under most conditions are fortunately definite, and are becoming more widely known and appreciated every year. The first and most important rule to be laid down is, *immediate operation if the case is diagnosed within twenty-four hours of the onset of the attack.* This rule should never be broken, for although operation cannot be said to be of vital necessity in every case, yet were one always performed, far fewer lives would be lost than at present. An attack which in the first twenty-four hours appears to be of a mild catarrhal type, may within a very few hours have assumed so grave a form that operation may be too late or at best a desperate resource. Nothing is more uncertain in these cases than the prognosis from day to day, one might almost say from hour to hour. Operation in the first twenty-four hours is little if at all more dangerous than an "interval" operation, as the appendix is not yet firmly embedded in a mass of adhesions, and can be readily removed. Should the diagnosis be uncertain or operation refused, the patient must be sat up in bed, not merely the head and shoulders raised by a few pillows, but the sitting position be insisted on; this is not difficult in the case of a child, and is of the utmost importance, since it ensures that any pus which may be formed will collect in the lower half of the abdomen, the iliac fossæ and pelvis, situations more easily accessible to surgery than the upper half of the abdomen. Of equal or greater importance is it to ensure absolute rest to the intestines, in order that protective adhesions, undisturbed by peristaltic movements, may form round the inflammatory focus. Therefore on no account give purgatives or enemata, methods of treatment usually the first to be adopted and responsible for the loss of many lives. For the same reason nothing must be given by the mouth, not even water. Continuous rectal saline infusion, or what answers the purpose equally well, and is less difficult of accomplishment, namely, ℥iv. to ℥v. saline given very slowly by an irrigator every two hours, will prevent thirst and also help in the elimination of toxins. Local treatment in the form of an ice-bag will do no harm and may be of service.

If the patient is not seen until the second or third day, the question whether to operate or not is often very difficult to decide. If improvement in the general condition is taking place, with a slightly lower temperature, decrease in pain, cessation of vomiting, and improving pulse, operation should be delayed, and the patient treated as mentioned above. A very strict watch, however, must be kept for any change for the worse, an increase in the rate of the pulse with decrease in volume being taken as imperative indications for immediate operation.

When once the presence of an abscess has been diagnosed, no treatment other than operation is to be considered, and the operation should be done at the first possible moment. In general peritonitis the necessity for operation is even greater than with an abscess.

The skin is better prepared by painting with tincture of iodine rather than by the usual methods of washing and scrubbing, the necessary manipulations of which are a source of danger by possibly causing the rupture of an abscess and the diffusion of its contents among the intestines.

Chloroform must on no account be used as the anæsthetic in these cases.

Until within the last two or three years it was a common experience at this hospital and elsewhere, to see children, in whom an appendix abscess had been opened and drained, die within twenty-four or forty-eight hours, apparently from an increase of toxæmia. These deaths we now know to have been due to the effect of chloroform poisoning, added to the toxæmia already present from the abscess. In this hospital, since the introduction of spinal anæsthesia, death from toxæmia following upon the simple opening and drainage of an abscess has disappeared, and our percentage of recoveries in cases of general peritonitis has also very greatly increased. With spinal anæsthesia no case is absolutely hopeless. If spinal anæsthesia is not available, ether, given by the open method, or, if the child is moribund, local anæsthesia, should be used.

### Abscess.

For abscess the operation performed should be the simplest possible consistent with a free evacuation of the pus and thorough drainage. The incision varies with the site of the abscess, and is best made over the most prominent part, or, if the abscess be in the iliac fossa or loin, a little to the outer side. The abscess is widely opened and carefully mopped dry, then a gentle examination with the finger is made to discover whether a stercolith or secondary pockets are present, but as little manipulation as possible is indulged in. On no account should a search for the appendix be made, though if this present itself in the cavity it should be removed. A very large drainage-tube, certainly not less than half an inch in diameter, should be used, but gauze packing should be avoided, as it is nearly always unnecessary, rapidly becomes sodden and swollen, and tends to prevent drainage. In the case of an abscess in Douglas' pouch, by far the best method of drainage is by means of an opening into the rectum, the drainage tube being brought out at the anus, supra-pubic drainage in this instance being very inefficient.

The after-treatment is precisely the same as that given above for a case that has not been operated upon. A little morphia may be given provided that there is no paralytic distension of the intestines. After forty-eight hours, if the case is doing well, small quantities of nourishment may be given by the mouth.

Complications in the form of secondary abscesses, if promptly recognised and dealt with surgically, though delaying recovery do not greatly add to the gravity of the prognosis. A fæcal fistula frequently develops, but seldom gives rise to trouble, usually closing spontaneously in from two to three weeks. Should a fistulous communication with the bowel persist, on no account be in a hurry to operate, as operation is always difficult and usually very dangerous. Also these fistulæ show a great tendency to spontaneous closure even after six to nine months.

### General Peritonitis.

Two main types of general peritonitis may be found, the serous or sero-purulent and the thick purulent form. The appendix itself is perforated or gangrenous, and not sealed off by adhesions, or may be lying in an imperfectly localised abscess. An incision is made over the appendix region and the appendix is removed, or if an abscess is present this is carefully mopped dry. Where the exudate is of the serous or sero-purulent type the pelvis and iliac fossa may be sponged dry, but it is not advisable to try and clean up the whole of the abdomen. Drainage tubes should be put into the right iliac fossa and into the pelvis.

When thick pus is found, an attempt should be made to remove as much as possible by means of mops on sponge holders, special attention being paid to the iliac and pelvic fossæ and to the two hypochondriac regions. Adequate drainage through multiple incisions must be provided, adherent lymph should not be removed from the intestines, and no attempt to wash out the peritoneal cavity should be made. After-treatment is the same as in the case of an abscess.

### Operation in the Quiet Stage.

No child should be allowed to have a second attack of appendicitis. Mildness of the first attack is no guarantee whatever that the second may not be fatal. It is argued by some that operation for the removal of the appendix is unnecessary in a case where there has been an abscess, as the appendix has probably been destroyed. This does no doubt sometimes occur, but I have seen several cases within the last few years where a child has had two, three, four, and even five attacks, each with an abscess, and in one case at any rate the last attack was fatal.

To recapitulate the treatment, if seen within twenty-four hours, operation. If not seen till later, be guided by the condition of the patient.

If no operation, sit the patient up in bed, give nothing by the mouth, above all no purgatives or enemata, but continuous rectal saline.

Lastly, when in doubt, operate.

